# Vectorial Capacity of *Aedes aegypti* for Dengue Virus Type 2 Is Reduced with Co-infection of *Metarhizium anisopliae*

**DOI:** 10.1371/journal.pntd.0002013

**Published:** 2013-03-07

**Authors:** Javier A. Garza-Hernández, Mario A. Rodríguez-Pérez, Ma Isabel Salazar, Tanya L. Russell, Monsuru A. Adeleke, Erik de J. de Luna-Santillana, Filiberto Reyes-Villanueva

**Affiliations:** 1 Laboratorio de Biomedicina Molecular, Centro de Biotecnología Genómica, Instituto Politécnico Nacional, Reynosa, Tamaulipas, Mexico; 2 Laboratorio de Inmunología Celular e Inmunopatogénesis, Departamento de Inmunología, Escuela Nacional de Ciencias Biológicas, Instituto Politécnico Nacional, Mexico City, Distrito Federal, Mexico; 3 Faculty of Medicine, Health and Molecular Sciences, James Cook University, Cairns, Queensland, Australia; 4 Public Health Entomology and Parasitology, Department of Biological Sciences, Osun State University, Osogbo, Nigeria; U.S. Army Medical Research Institute of Infectious Diseases, United States of America

## Abstract

**Background:**

*Aedes aegypti*, is the major dengue vector and a worldwide public health threat combated basically by chemical insecticides. In this study, the vectorial competence of *Ae. aegypti* co-infected with a mildly virulent *Metarhizium anisopliae* and fed with blood infected with the DENV-2 virus, was examined.

**Methodology/Principal Findings:**

The study encompassed three bioassays (B). In B1 the median lethal time (LT_50_) of *Ae. aegypti* exposed to *M. anisopliae* was determined in four treatments: co-infected (CI), single-fungus infection (SF), single-virus infection (SV) and control (C). In B2, the mortality and viral infection rate in midgut and in head were registered in fifty females of CI and in SV. In B3, the same treatments as in B1 but with females separated individually were tested to evaluate the effect on fecundity and gonotrophic cycle length. Survival in CI and SF females was 70% shorter than the one of those in SV and control. Overall viral infection rate in CI and SV were 76 and 84% but the mortality at day six post-infection was 78% (54% infected) and 6% respectively. Survivors with virus in head at day seven post-infection were 12 and 64% in both CI and SV mosquitoes. Fecundity and gonotrophic cycle length were reduced in 52 and 40% in CI compared to the ones in control.

**Conclusion/Significance:**

Fungus-induced mortality for the CI group was 78%. Of the survivors, 12% (6/50) could potentially transmit DENV-2, as opposed to 64% (32/50) of the SV group, meaning a 5-fold reduction in the number of infective mosquitoes. This is the first report on a fungus that reduces the vectorial capacity of *Ae. aegypti* infected with the DENV-2 virus.

## Author Summary

Dengue is a worldwide public health problem. There is not an effective vaccine yet; the chemical struggle against its transmitter, the mosquito *Aedes aegypti*, is onerous and erratic, and the community participation to eliminate vector breeding sites is unconfident. Here, we examined mosquitoes fed on human blood mixed with the Dengue virus, by exposure to the fungus *Metarhizium anisopliae*, to test whether the fungus halts the viral dissemination from midgut to head in co-infected (CI) insects. We found an overall viral infection rate in CI mosquitoes of 76% but infected or not, most (78%) died before or at day six post-infection; only six (12%) out of 50, survivors had virus in head and were potentially infectious at day seven post-infection. A higher infection (84%) was observed in single-virus infected mosquitoes, but they suffered only 6% mortality after 6 days and 32 (64%) survivors tested positive for virus in head after 7 days. Survival, fecundity and ovaric cycle of CI mosquitoes were reduced in 70, 52 and 40% in comparison to the ones of control. Therefore, if the fungus caused a 5-fold reduction in the number of infectious mosquitoes, it has potential to be evaluated against the Dengue transmitter in field.

## Introduction

The susceptibility of *Aedes aegypti* adults to infection with *Beauveria bassiana* was first reported in the late 1960s [1]. However the potential of entomopathogenic Ascomycetes (Hypocreales) as adulticides of vector mosquitoes was largely overlooked until *Metarhizium anisopliae* was demonstrated to induce mortality of *Culex quinquefasciatus* and *Anopheles gambiae* [2]; and sequentially both *M. anisopliae* and *B. bassiana* have been tested against *Ae. aegypti* and *Aedes albopictus* [3]. The successful infection of adult female mosquitoes has been made via direct contact [4], [5] and also via auto-dissemination from males to females when mating [6], [7].

The increasing interest in exploring these fungi as biocontrol agents of dengue vectors stems from the fact that they are ubiquitously available, relatively cheap to mass-produce, and kill mosquitoes effectively [8]. In addition to the infection studies, attention has also been focused on other topics such as determining their safety to public health [9], and the effect of different surfaces on the infectivity of conidia to resting mosquitoes [10]. Likewise, some devices with inoculum baited with lures have also been tested for attracting and infecting adults to avoiding domiciliary sprayings [11].

*Metarhizium anisopliae* pathogenesis to insects has been widely documented [12]. The fungus is hemibiotrophic [13]. Conidia germination and cuticle perforation last around 24 hours [14]. After penetration, the pathogen produces hyphal bodies or blastospores invading the whole host's hemocele, depleting nutrients and killing the insect by starvation, dehydration, and toxemia [15]. It is therefore proposed that the rapid fungal invasion could affect the survival of the DENV virus if both are present in the same female of *Ae. aegypti*, weakening its vectorial competence. Here, we fed *Ae. aegypti* females with DENV-2-infected human blood, and/or exposed them to *M. anisopliae* conidia to produce single-fungus (SF), single-virus (SV) and co-infected (CI) mosquitoes. The parameters evaluated included mosquito survival, fecundity and first gonotrophic cycle (GC) length, plus the viral infection rate in the midgut and head.

## Materials and Methods

### Cell lines, virus titers, fungus and mosquito infection

The DENV-2 Yuc 18500 strain was isolated from blood of a sick person at Merida city in 2008; it is deposited at the “Collection of Arboviruses isolated at the Yucatán Peninsula” of the Regional Research Center “Dr Hideyo Noguchi”, University of Yucatan (UADY), Mexico; its use in this study was approved by written consent given by Dr. Fernando Andrade-Narvaez, Chair, Bioethics Committee of the Regional Research Center “Dr Hideyo Noguchi”, University of Yucatan (UADY), Merida, Yucatan, Mexico. In addition, all members of the Bioethics Committee provided informed consent. This strain was used to infect C6/36 *Aedes albopictus* cells at a starting viral titer of 1.5 particles/cell. C6/36 cells were grown in Leibovitz's medium but the infected ones were held in medium containing 2% fetal bovine serum. The Ma-CBG-1 strain of *M. anisopliae* was isolated from soil collected at rural habitats around the city of Saltillo; cultured on potato-dextrose-agar; and passaged three times through living hosts (*Ae. aegypti* females) before the study. At 12 days post-infection (PI) the virus was harvested. Mosquitoes used in the study were derived from a colony of *Ae. aegypti* that was established in 2008 with larvae collected in Monterrey, México. Four to seven day-old female *Ae. aegypti* mosquitoes were exposed to single infections of either Ma-CBG-1 *M. anisopliae* strain at 1.6×10^8^ conidia mL^−1^ (SF) or DENV-2 (SV); and both fungus and virus (CI). Fungal infections were done as described previously [7]. For the virus infections, females were confined in 1-liter glass flasks, and were fed on 2,320 µL defibrinated human blood and 680 µL of virus suspension containing a titer of 1×10^7^ plaque-forming units (PFU) mL^−1^, for 1 hour via a water-jacketed membrane feeding apparatus [16]. Control mosquitoes (C) and those in the SF group were fed with non-virus infected blood in the same manner. After blood feeding, mosquitoes were anaesthetized by exposing the flask to 4°C for 25 minutes, and then only blood-fed females were transferred to the holding containers for each bioassay.

### Bioassays

Three bioassays (B) were conducted. In B1, the survival of *Ae. aegypti* females was compared between each treatment: CI, SF, SV, and control. Fifty mosquitoes per treatments were used, encompassing two replicates of 25 each in a 1-liter plastic flask. The 25 females per replicate were randomly selected from, those emerged from larvae (200 larvae/liter) of the same plastic tray and all replicates were conformed by adults emerged from larvae of different plastic trays, origin (eggs) and handling. Dead insects in treatments were recorded and removed daily. The cadavers were submerged twice in 1% chlorine solution, washed in distilled H_2_O, and placed in humid chambers for conidiation. This bioassay was run until the last insect died.

In B2 the viral infection rate in the midgut and head of mosquitoes was examined. Treatments were CI and SV, with also fifty insects per treatment and two replicates as in B1. Dead females were registered daily until six days PI without registering sporulation. At day 7 PI all surviving mosquitoes were cold-killed. Day 7 was the cut-off point because this is the average extrinsic incubation period (EIP) for the DENV-2 in *Ae. aegypti* [17], [18] and these studies were conducted at 30 (±2)°C. Mosquito midguts and heads were dissected on a glass slide containing 10 µL of phosphate-buffered saline; then were fixed and placed into 0.2 mL Gold-PCR tubes containing 150 µL of 4% paraformaldehyde and kept at 4°C until further analysis.

In B3, sixty females were used per treatment to assess the impact of fungal infection on fecundity and length of the first GC. The same treatments as in B1 were set up but with three replicates of 20 females each. However here, the mosquitoes of each replicate were individually separated into 40-ml capped-vials, containing a small amount of water and cardboard to record daily oviposition for each individual female.

### RT-PCR and immunofluorescent assay

In B2, the viral infection rate in the females sacrificed at 7 days PI, as well as in cadavers collected at 1–6 days, was determined using PCR. Details of protocols for viral RNA purification, cDNA synthesis, primer sequences, PCR conditions, and detection of PCR products by agarose gel electrophoresis, were reported earlier [16]. The preparation of midguts and heads, the immunofluorescent assay, and stain of female's tissues have also been published elsewhere [18].

### Statistical analysis

Daily mortality rate was used to compute the median lethal time (LT_50_) per treatment with the Kaplan-Meier model; the model was stratified by replicate number to account for dependencies for mosquitoes which were held within each replicate. In B2, two 2×3 cross tabulation analyses by χ^2^ using Fisher's exact test were applied to the percentages of females with viral infection in the midgut, then with disseminated infection in head and non-DENV-2-infected, across the CI and SV treatments; the first one was an overall analysis while the second was only for the three groups of surviving females across both treatments on 7 days PI. The Fisher's exact test was used because the sample sizes were small. In B3, a 2×2 cross tabulation analysis by χ^2^ was applied to the percentages of ovipositing females in both CI and SV treatments; moreover, a one-way analysis of variance was applied for fecundity and GC; means were contrasted with a Ryan test. All analyses were performed with SAS [19].

## Results

**Figure 1:**
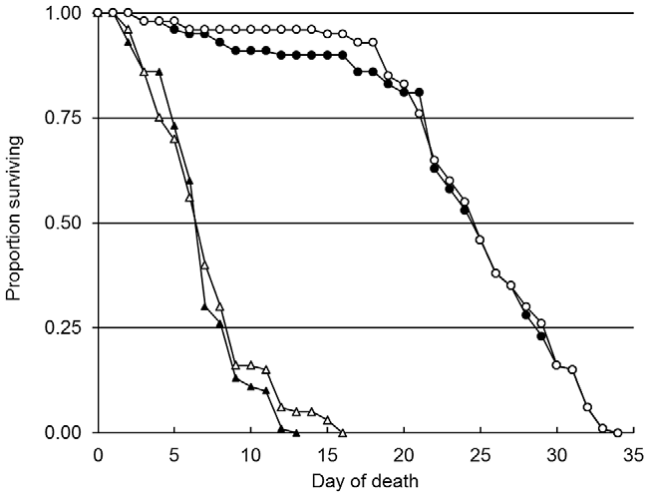
Survival curves for four experimental groups of *Ae. aegypti* females (n = 50), based on the daily probability values computed by the Kaplan-Meier model for Bioassay 1. Mosquitoes were challenged with either the DENV-2 (1×10^7^ PFU/mL) (SV = black circle, CI = black triangle) and/or exposed for 24 hours to 1.6×10^8^ conidia mL^−1^ of *M. anisopliae* (SF = white triangle); controls (white circle) were exposed to neither treatment.

In B1, the overall survival varied among the four groups (χ^2^ = 237.25, df = 3, p<0.0001); further analysis indicated that there was no statistical difference in the survival of DENV-2 infected (CI) and non-DENV-2-infected *Ae. aegypti* females (SF) (χ^2^ = 2.87, df = 1,p>0.05). The LT_50_ of fungal infected females was 6.93 (range, 6.59–7.27 days, 50 samples) for CI and 7.23 (range, 6.80–7.66 days, 50 samples) for SF. The same occurred for the survival of females infected only with the virus (SV) which was similar to the uninfected controls (χ^2^ = 0.21, df = 1, p>0.05). The LT_50_ for SV and control mosquitoes was 24.00 (range, 23.08–24.98 days, 50 samples) and 24.83 (range, 24.06–25.60 days, 50 samples) days respectively. Overall, *M. anisopliae* reduced the survival (as indicated by the LT_50_ values) of both CI and SF mosquitoes by ≈70% (Figure 1). The sporulation rates of cadavers collected from the CI and SV treatments was 85% on average for both experimental factors. Therefore, regardless of the virus, the fungus killed 85% of mosquitoes in both treatments.

**Figure 2:**
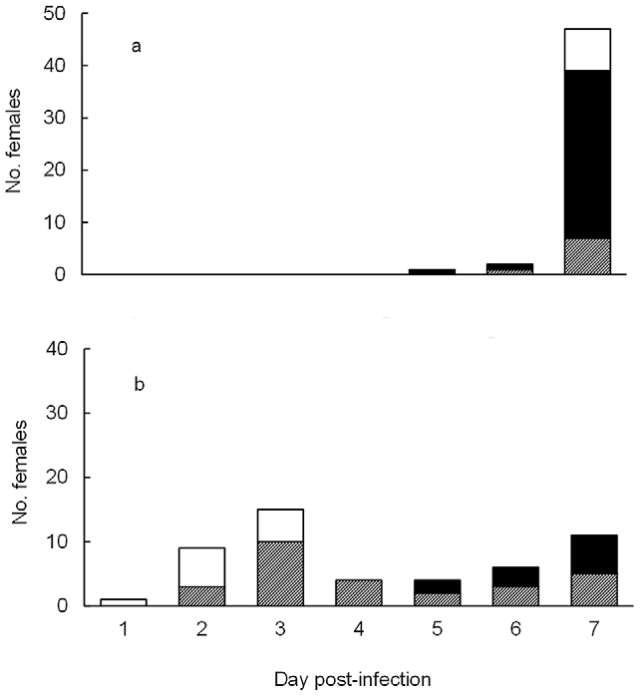
Viral infection rate in midgut and head. Number of *Ae. aegypti* (50 per treatment) virus-free females (white square), with the DENV-2 virus in midgut (grey square) and in both midgut and head (black square) that died at different days until the day 7 PI when survivors were sacrificed and tested for viral presence. 2a: Mortality for SV mosquitoes. 2b: Mortality for CI females.

In B2 at 7 days PI, the mortality rate of SV females was only 6% (3/50; Figure 2a), while in CI mosquitoes there was 78% (39/50; Figure 2b) mortality. For the SV treatment after 7 days, the percentage of surviving mosquitoes with virus in the midgut, head or non-DENV-2-infected was 15% (7), 64% (32) and 17% (8) respectively. For the CI treatment after 7 days, the percentage of surviving mosquitoes with virus in the midgut, head and non-DENV-2-infected was 10% (5), 12% (6) and 0% (0) respectively. As such, for mosquitoes surviving to 7 days PI, there was a reduced proportion which were able to develop a head infection between the CI and SV groups (χ^2^ = 6.14, df = 2, p<0.05). However, an assessment of vectorial capacity should also account for mortality. When mortality is considered, 64% (32/50) of the mosquitoes in the SV treatment were alive and potentially able to transmit at 7 days PI; compared with only 12% (6/50) in the CI group (χ^2^ = 17.99, df = 1, p<0.0001). Therefore, there was a 5-fold reduction in the number of potential infective females due to the high mortality of females before they were able to complete the EIP.

**Figure 3:**
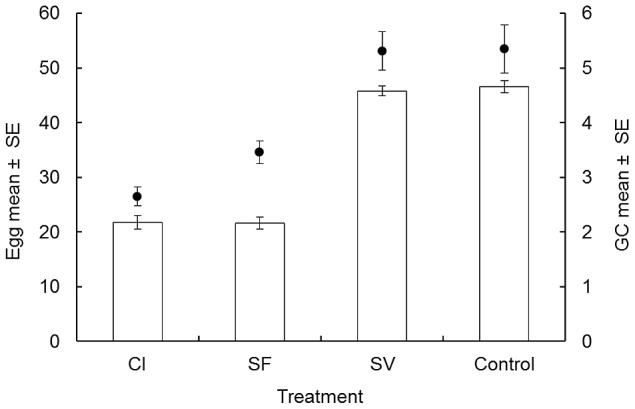
Effect of the fungus on reproduction. Impact of infections with *M. anisopliae* and/or the DENV-2 on the number of eggs per female (bars), and duration of the gonotrophic cycle (GC) in days (black circles) of *Ae. aegypti* females after exposure to CI, SF, SV treatment plus control (three replicates, 20 females each per treatment). CI and SF were not different significantly, but both were lower than SV and Control.

In B3, the percentage of fungus-infected individuals which oviposited within 7 days was only 56% (23/41) for CI and 42% (18/42) for SF. This was ≈50% less females than observed for the non-fungus treatments where 100% of females oviposited (SV = 59/59 and Control = 59/59 (χ^2^ = 294.00, df = 3, p<0.0001). Of the females which laid at least one egg, fungus-infected females generally laid less eggs. The mean number of eggs per female for fungal-infected treatments were 21.75 (range, 19.10–24.40 eggs, 20 samples) in CI and 21.65 (range, 18.19–25.11 eggs, 20 samples) in SF, contrasting with the non-fungus treatments where the mean number of eggs per female was 45.76 (range, 40.45–51.07 eggs, 20 samples) in SV and 46.58 (range, 41.23–51.93 eggs, 20 samples) in CI. Therefore, the fecundity of *Ae. aegypti* was reduced by 52% (Figure 3) (F = 22.95, df = 3, p<0.001). The infection of females with *M. anisopliae* was also observed to accelerate the oogenesis. The GC of fungus-infected females was 2.65 (range, 2.48–2.82 days, 20 samples) in CI and 3.46 (range, 3.25–3.67 days, 20 samples) in SF and both of these were shorter than the GC of SV and uninfected mosquitoes which were 5.31 (range, 4.96–5.66 days, 20 samples) and 5.35 (range, 4.91–5.79 days, 20 samples) days respectively (F = 14.15, df = 3, p<0.001). Thus, the fungal co-infection diminished the length of the GC by 40% (from 5 to 3 days).

## Discussion

**Figure 4:**
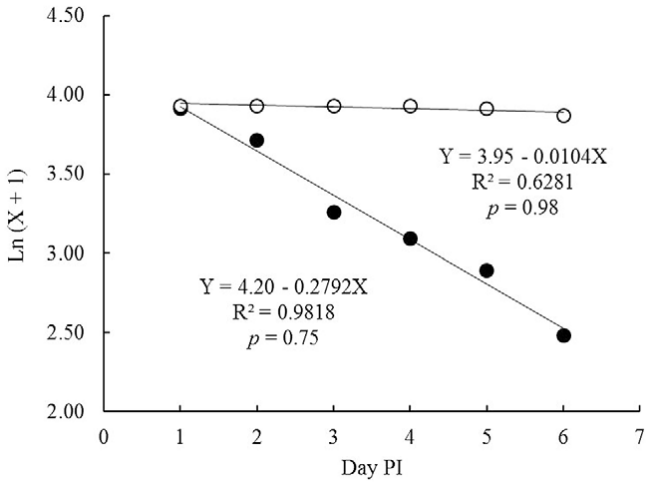
Daily survival probability (*p*) for *Ae. aegypti* females in both SV (white circle) and CI (black circle) treatment computed by regression of number of females died daily [ln (X+1)] on the day PI.

In *Ae. aegypti* co-infected with DENV-2 and with a Mexican strain of *M. anisopliae* there was a 5-fold reduction in the number of mosquitoes which survived the EIP and with potential to transmit dengue. The most sensitive component of vectorial capacity (*C*) as defined by the Ross-MacDonald model is daily survival [20], [21]. *M. anisopliae* infection killed the majority of females (78–88%) before surviving the 7-day EIP. Furthermore, of the fungus-infected mosquitoes which did survive the EIP, there was a reduced chance of them becoming infectious after a feeding on DENV-2 infected blood. To express more clearly the impact of the fungus on *C* of the dengue vector, we computed this index taken our own data and others from literature: Daily survival probability (*p*) for SV and CI females was computed by regressing the number of survivors [ln (X+1)] on days PI up to day 6, and were 0.98 and 0.75 with determination coefficients of R^2^ = 0.62 and 0.98, respectively (Figure 4). These different rates mean a reduction in *p* of 0.23 by the fungal effect. The *C* model *ma* ^3^ *bp* ^n^/-ln(*p*) and their components are defined as follows: *m* = “daily biting rate”, *a* ^3^ = “the human biting rate” powered to the number of blood meals per GC, which is at least three for *Ae. aegypti* [22]; *a* = 1/GC, *b* = proportion of infectious females at the EIP which were 0.64 and 0.12 for SV and CI groups, respectively, and *p* ^n^/-ln(*p*) = the expected infective life in days after the EIP. Now in Monterrey, MX, the *Ae. aegypti* annual population is bimodal with the highest peak in October, where a time series of 19 consecutive days of human-landing captures allowed to estimate a *m* = 37 bites/human/day [23]; then keeping constant *m* = 37, *a* = 1/GC days (5 days for SV and 3 for CI) and *n* = EIP = 7 days, the calculation results in a *C* = 8.14 and 0.07 for SV and CI, respectively; this means that the fungus reduced the *C* by 116 times in CI compared to the one of SV-infected females. This would be a drastic impact of *M. anisopliae* on the vectorial capacity of *Ae. aegypti* in field.

Comparatively, the 64% of females that fed on DENV-2 infected blood and became infectious within 7 days, is two-fold superior than 27% and 30% recorded in *Ae. aegypti* from Texas, USA, and Chiapas, Mexico infected with the DENV-2 Southeast Asia strain, respectively [24], but is similar to the competence of *Ae. aegypti* from Australia [25].

The fungus was able to quickly invade the tissues and cells causing the early death of CI mosquitoes before many of them were able to survive the entire EIP. This observation is supported by previous work which detected *M. anisopliae* hyphal bodies circulating in the hemolymph of *Locusta migratoria manilensis* at day 2 PI [26]. Generally cuticle perforation and hemocele invasion last around 20 hours [14]. Once the host is invaded, the fungus starts to consume nutrients and propagate. By day 2–3 PI the fungus releases detectable levels of toxins and enzymes [27], [28], compromising the immune system of the mosquitoes triggering mortality, as was observed in CI and SF females by 3 days PI. Similarly, this competition for nutrients between the host and fungus, could also explain the 52% reduction in fecundity and a shortening of the GC from 5 to 3 days in B3. In a previous study, we conducted with this fungus but with a highly virulent strain (Ma-CBG-2) found that in mycosed females the fecundity was reduced to almost zero [7]. In addition, the *Anopheles gambiae* female mosquitoes tend to take smaller blood meals after becoming fungus-infected [29], [30] and this also plausibly explains the reduced number of females which actually laid eggs. In the current experiments, we controlled for any possible biases of dengue infection on fecundity by using the four experimental factors (CI, SV, SF and control). It is a general knowledge that entomopathogens usually induce changes in vector behavior and physiology [31], [32]. The shorten of GC observed in B3 could be part of an adaptive strategy by the female mosquitoes in achieving reproduction before the pathogens drastically deplete the nutritive resources required for the egg development. It could also be an adaptive behavior by the mosquitoes which are unlikely to survive the virulence of the pathogens as earlier observed in crickets infected with bacteria and parasites [33], [34], while the oogenesis of *Schistocerca gregaria* and *Ae. aegypti* was speeded up by *M. anisopliae* and *B. bassiana* [35], [36]. However, instead of curtailment of reproductive potential of the insects, some pathogens have been known to enhance the reproductive success of their host through higher production of offspring early in life; a phenomenon often referred as “fecundity compensation” [37]. These reactions may partly be mediated by the immune system of the insects in response to the infection as earlier posited by researchers [33], [37].

Concerning the impact of the virus, no effect of dengue infection on fecundity was noted, contrary to previous research. There is some evidence that the dengue virus exerts low mortality rates on *Ae. aegypti* adults, as well on its fecundity, as was recently reported for Brazil, where 4–5 day-old *Ae. aegypti* females were fed with rabbit blood mixed with 3.6×10^5^ PFU mL^−1^ of the DENV-2 (strain 16681) [38]. The authors found that the longevity of infected mosquitoes averaged 26 days, which is similar to the 24 days observed here in SV females. They reported a viral infection of 66% which is also similar to the 68% (6 females with virus in head and 5 with virus in midgut) obtained in SV females at 7 days PI that we found; the viral effect on fecundity was examined by a logistic regression; however the authors only reported that the mean number of eggs per GC through five GCs tended to be lower in infected mosquitoes, without mentioning the specific means. The reduced fecundity may be beneficial for mosquito control because the population size of consecutive generations will be proportionally smaller; however this reduction occurred concomitantly with a shorter GC, which not necessarily implies only a faster reproduction. A shorter GC may also alter the natural mortality of populations by increased exposure to predation and other adverse environmental factors when search more often for blood-meals. It is possible that the direct mortality observed in our laboratory study may be compounded such indirect increases of mortality under field conditions.

In conclusion, *M. anisopliae* has the potential to drastically affecting the vectorial capacity of *Ae. aegypti* in the field and could be accomplish without necessarily using a highly virulent strain (LT_50_ = 3–4 days) of the fungus. Whether these conditions could be fulfilled in field is the aim for an ongoing investigation by our team.
